# Rapid Assessment of Italian Honey Chemical Composition and Botanical Origin Using NIR Spectroscopy Coupled with Chemometric Analysis

**DOI:** 10.3390/s26092796

**Published:** 2026-04-30

**Authors:** Alessia Zoroaster, Andrea Calore, Anisseh Sobhani, Nicoletta Dainese, Anna Granato, Severino Segato, Lorenzo Serva

**Affiliations:** 1 Department of Animal Medicine, Production and Health, University of Padova, Viale dell’Università 16, 35020 Legnaro, Italy; alessia.zoroaster@unipd.it (A.Z.); andrea.calore.5@studenti.unipd.it (A.C.); anisseh.sobhani@studenti.unipd.it (A.S.); severino.segato@unipd.it (S.S.); 2 NRL for Honey Bee Health, Istituto Zooprofilattico Sperimentale delle Venezie, Viale dell’Università 10, 35020 Legnaro, Italy; ndainese@izsvenezie.it (N.D.); agranato@izsvenezie.it (A.G.)

**Keywords:** portable systems, food authentication, non-destructive analysis, food quality control, spectral data processing, machine learning, rapid screening

## Abstract

**Highlights:**

**What are the main findings?**

- NIR analytical configurations exhibit system-dependent performance.
- A benchtop setup achieves the highest predictive accuracy.
- Spectral resolution and signal-to-noise ratio affect model performance.

**What are the implications of the main findings?**

- A portable NIR-based setup enables moderate accuracy, suitable for rapid field screening.
- Wavelength selection adds little accuracy, but spectral quality is more critical.

**Abstract:**

Honey quality and authenticity assessment require rapid and reliable analytical tools capable of supporting both laboratory and on-site applications. Near-infrared (NIR) spectroscopy represents a non-destructive and cost-effective approach; however, its performance depends on instrument characteristics and chemometric strategies. This study compared one benchtop and two portable NIR-based systems for predicting key physicochemical parameters (moisture, electrical conductivity, glucose, fructose, reducing sugars, pH, hydroxymethylfurfural, and diastatic activity) and for discriminating botanical origin in 80 Italian honey samples. Spectral data were processed using multiple pre-processing techniques and algorithms (PLS, k-NN, Random Forest, SVM), with and without wavelength selection (siPLS and CARS-PLS), under cross-validation schemes. The benchtop system achieved the highest regression performance (R^2^ up to 0.91 for glucose and electrical conductivity) and the most reliable botanical classification (balanced accuracy = 0.90). Portable systems showed moderate predictive ability for bulk compositional parameters (R^2^ up to 0.86 for glucose) but limited classification performance. Wavelength selection resulted in only marginal improvements. Hydroxymethylfurfural and diastatic activity were poorly predicted (R^2^ up to 0.49), likely due to their low concentrations. Summarising, the main outcomes suggested that tested portable NIR settings are also suitable for rapid quantitative screening of chemical traits, whereas the benchtop system provide higher precision for botanical qualitative authentication.

## 1. Introduction

Honey is a chemically complex natural matrix whose physicochemical characteristics are strongly influenced by botanical and geographical origin, determining its compositional variability, sensory profile, and market value [1,2]. Indeed, depending on the geographical and botanical origin, the market appeal of honey is influenced by several factors, including climatic, soil, and floral conditions, resulting in a wide range of nutritional and rheological traits and leading to fluctuations in overall quality and consumer acceptance [3]. Therefore, to safeguard the labelled quality and to claim the presence of beneficial substances [4], it is necessary to develop accessible, accurate, and rapid technologies capable of predicting the main chemical characteristics, such as sugar composition, physical parameters, and indices of stability during storage.

Beyond intrinsic variability, heat during processing and storage promotes chemical degradation, including hydroxymethylfurfural (5-HMF) formation and moisture redistribution, increasing heterogeneity in crystallised and biphasic honey samples [2]. Additionally, adulteration—namely the deliberate incorporation of low-cost sweeteners or syrups to increase product volume—represents a critical challenge. Such practices distort compositional profiles and compromise regulatory compliance, further increasing analytical complexity and undermining consumer trust and market transparency [5,6]. Overall, such alterations can be detected by near-infrared spectroscopy (NIR), enabling discrimination between untreated, heat-treated, and adulterated samples, although partial spectral overlap may occur under extreme processing conditions [7,8,9]. For this reason, NIR spectroscopy is emerging as a rapid, non-destructive, and cost-effective tool for honey analysis [5,10,11]. However, the potential of NIR depends on appropriate data pre-processing and chemometric modelling, which enable the extraction of meaningful information from complex spectral signals. Within this framework, multivariate techniques such as principal component analysis (PCA), partial least squares (PLS) regression, artificial neural networks (ANNs), and support vector machines (SVMs) have been widely applied for adulteration detection and the discrimination of honey samples according to botanical and geographical origin [5,11,12]. The selection of spectral pre-processing strategies is data- and goal-dependent, and robust results require appropriate model validation [13,14]. Furthermore, the robustness and real-world applicability of advanced chemometric strategies require careful estimation under industrial conditions, where cost-effectiveness, model stability, and operational feasibility are key constraints [10].

A comprehensive review by [15] explored the utility of NIR spectroscopy as a non-destructive analytical method for concurrently evaluating honey quality and authenticity, with a specific focus on key parameters such as sugar content, moisture levels, and 5-HMF content. That review, together with a parallel systematic analysis of FTIR applications by [16], confirmed that vibrational spectroscopy is a reliable technique for honey characterisation without cumbersome sample preparation, with spectral differences attributed to sugars and water enabling differentiation of honey types and detection of adulteration, provided that advanced data analysis is applied. On the specific front of botanical origin, ref. [17] demonstrated that a portable FT-NIR approach combined with chemometrics can discriminate multifloral and unifloral honey samples by botanical and geographical origin with 90% and 95% accuracy, respectively, while simultaneously detecting syrup adulteration; multiplicative scatter correction was found to be the most effective preprocessing strategy among those tested. In parallel, ref. [15] identified a set of persistent structural limitations across the field: a large proportion of published models rely exclusively on internal cross-validation, lacking independent external testing or interlaboratory ring trials essential for verifying robustness under realistic variability, while standardised spectral acquisition protocols and shared spectral libraries are identified as critical prerequisites for regulatory deployment.

A recent review by Prata and Da Costa (2024) [15] highlights that analytical performance in vibrational spectroscopy is strongly influenced not only by the underlying instrument technology but also by the measurement configuration, including acquisition mode and pre-processing strategies. While benchtop systems are often associated with higher accuracy and stability, and portable devices with rapid on-site screening capabilities, the review also emphasises that predictive performance and model robustness are highly dependent on the overall analytical setup. Importantly, it identifies persistent limitations in the field, including the lack of standardised acquisition protocols and controlled cross-platform studies, which hinder direct comparability between systems. These gaps motivate the system-level approach adopted in the present work. Against this backdrop, Italy represents a particularly relevant and understudied case. Italy is one of the foremost honey-producing countries in Europe, with a recognised diversity of unifloral varieties—including acacia, chestnut, citrus, dandelion, linden, rhododendron, and sunflower—whose authentication holds significant commercial and regulatory value under the recently revised EU Directive 2024/1438 [18].

The present study addresses these gaps by systematically evaluating three NIR instrument configurations, along with multiple pre-processing strategies and regression algorithms, on a dataset of authenticated Italian honeys. This approach is designed to generate evidence relevant to both the specific characterisation of Italian honey and the broader challenge of instrument-independent, transferable NIR calibration for honey authentication.

Based on the analysis of eighty Italian honey samples, the present study evaluated the performance and robustness of different NIR-based analytical systems—comprising instrument configuration, acquisition geometry, spectral pre-processing, and modelling strategy—under realistic conditions for honey quality and authenticity assessment. The three analytical systems were based on one benchtop and two portable NIR systems for the prediction of key honey chemical traits (pH, moisture, electrical conductivity, glucose, fructose, reducing sugars, hydroxymethylfurfural, and diastatic activity), evaluating different combinations of spectral pre-processing strategies and modelling algorithms. The same framework was applied with and without targeted wavelength (band) selection to assess its impact on model robustness and predictive performance. In parallel, the ability of the three NIR-based systems was investigated under the same comparative conditions to discriminate botanical origin (polyfloral, chestnut, and pooled multifloral class). Overall, the study aimed to support the development of reliable, transferable NIRS-based tools for laboratory- and on-site assessment of honey quality and authenticity.

Finally, the effect of the botanical group on the chemical composition was further examined.

## 2. Materials and Methods

### 2.1. Sample Collection

Eighty Italian honey samples collected in June 2024 from three regions of southern Italy—approximately 39–41° N, 14–17° E—were provided by the Consorzio Nazionale Apicoltori (CONAPI), headquartered in Monterenzio (Bologna, Italy). The dataset comprised two main botanical categories, including 30 polyfloral (PF) and 30 chestnut (CH) honey samples. To increase spectral variability for botanical origin clustering analysis, additional honey types were included: acacia (AC, *n* = 4), citrus honeys (CF, *n* = 4; including orange, clementine and lemon blossom), honeydew (HO, *n* = 5), French honeysuckle (FH, *n* = 2), and linden (LN, *n* = 3). Botanical origin was determined by melissopalynological analysis and certified by CONAPI, in accordance with current European honey legislation [19]. Each sample consisted of 150 g of unfiltered and unpasteurized honey. Samples were stored under refrigerated conditions (4 ± 2 °C) and analysed within 60 days of collection. All wet-chemistry analyses were conducted at the Istituto Zooprofilattico Sperimentale delle Venezie (Legnaro, Italy), while NIR spectral analyses were performed at LabCNX of Padova University.

### 2.2. Analytical Procedures and Spectral Acquisition

The physicochemical properties of the honey samples were determined according to the official methods established by Ministerial Decree [20], and the evaluated parameters comply with Italian Legislative Decree [19]. In particular, moisture content was measured using a Mettler Toledo RE 40 digital refractometer (Mettler Toledo, Columbus, OH, USA) and calculated according to the Wedmore equation, while electrical conductivity and pH were determined after dilution in bidistilled water using a Mettler Toledo Excellence T50 conductivity titrator (Mettler Toledo, Columbus, OH, USA) and a SevenCompact™ S220 pH meter (Mettler Toledo, Columbus, OH, USA), respectively. Diastatic activity was evaluated spectrophotometrically using the PHADEBAS^®^ Honey Diastase Test (Phadebas, Kristianstad, Sweden) with absorbance measured at 620 nm using a SpectraMax^®^ ABS UV–Vis spectrophotometer (Molecular devices, San Jose, CA, USA). Glucose, fructose, sucrose and 5-HMF contents were quantified by high-performance liquid chromatography (HPLC) using, respectively, a Shimadzu LC-20 AD system (Shimadzu, Kyoto, Japan) equipped with a refractive index detector and an Agilent 1290 Infinity II system (Agilent, Santa Clara, CA, USA) equipped with a UV detector set at 285 nm. Chromatographic separation was performed on an aminopropyl column (apHera™ NH_2_, Merck KGaA, Darmstadt, Germany) under isocratic acetonitrile–water elution for sugars and on a reversed-phase C18 column under isocratic water–methanol elution for 5-HMF.

For NIR analysis, approximately 3 g of each sample was analysed in transflectance mode using a benchtop scanning monochromator FOSS DS-2500 (FOSS Analytical A/S, Hillerød, Denmark), operating in the 850–2500 nm spectral range with a resolution of 0.5 nm, further referred to as “benchtop system”. Spectra were collected using a slurry cup equipped with a quartz window (12.6 cm^2^) and a 0.5 mm optical-path gold reflector and recorded as absorbance (log(1/R)) using WinISI software (version 4.10.0.15326; FOSS Analytical A/S). Portable NIR measurements were subsequently performed on the same samples: a NeoSpectra™ spectrometer (Si-Ware Systems, Menlo Park, CA, USA), operating in the 1350–2500 nm range with a spectral resolution of 16 nm in transflectance mode with a scan time of 2 s, further referred to as “portable wide-range system “, and an AlbaNIT spectrometer (GraiNit s.r.l., Padova, Italy), covering the 1102–1600 nm range with a spectral resolution of 2 nm, used in transmittance mode with an 8 mm path glass cuvette (Avantor™, VWR, Radnor, PA, USA), further referred to as “portable narrow-range system”. Spectra from the latter were acquired over 34 s and averaged over twenty consecutive scans. For all instruments, to reduce crystallisation and ensure sample homogeneity, honeys were heated at 30 °C for 30 min before analysis; this temperature was reported to be adequate to preserve honey quality. Moreover, each sample was scanned in duplicate using independent aliquots, and the average spectra were further used [7]. These differences in configuration resulted in a comparison across distinct instrumental settings rather than a direct instrument-to-instrument comparison.

For simplicity, the terms ‘benchtop’ and ‘portable’ systems are used throughout the manuscript; however, they should be interpreted to refer to complete analytical configurations, including the specific instrument, acquisition geometry (e.g., transmittance or transflectance), and measurement setup, rather than to the instrument class alone.

### 2.3. Standards and Analytical Validation

The quality control of the analytical methods was ensured through calibration procedures, precision assessment, and the use of certified reference materials (CRMs), in accordance with the official method [18]. To verify the refractometer calibration for moisture content determination, two solutions with known refractive indices were used: dodecane (C_12_H_26_; LGC Standards, Teddington, UK) and 2,4-dichlorotoluene (C_7_H_6_Cl_2_; LGC Standards, Teddington, UK). The operating range for moisture determination was 13–25%, and method precision, expressed as relative standard deviation (RSD, %), ranged between 1.05% and 1.21%. For electrical conductivity determination, the calibration of the titrator was verified using two standard solutions prior to sample analysis: a 0.001 M KCl reference solution and a 0.01 M KCl reference solution (Merck KGaA, Darmstadt, Germany). The expanded uncertainty associated with electrical conductivity measurements was estimated at 7.2% of the measured value. For diastatic activity determination, a certified reference material (T2858QC; Fapas^®^, York Biotech Campus, Sand Hutton, UK) was used to assess method accuracy and reliability. The limit of detection (LOD) was 0.9 Schade units, while method precision, expressed as RSD (%), was 4.83%. For sugar determination, glucose, fructose, and sucrose (Merck KGaA, Darmstadt, Germany) were used to construct the calibration curve. In addition, a certified reference material (T2858QC; Fapas, York Biotech Campus, Sand Hutton, UK) was analysed to assess method accuracy and reliability. The working range was 10–50% for glucose and fructose and 2–10% for sucrose, while method precision (RSD, %) was 3.57% for glucose and fructose and 12.5% for sucrose. Finally, for HMF determination, 5 hydroxymethylfurfural (97.5% purity; Merck KGaA, Darmstadt, Germany) was used to construct the calibration curve, while a certified reference material (T2859QC; Fapas, York Biotech Campus, Sand Hutton, UK) was used to evaluate method accuracy and reliability. The operating range was 1–60 mg/kg, and method precision (RSD, %) ranged between 1.77% and 2.75%. The relative standard deviation (RSD, %), evaluated under repeatability conditions, was determined at different concentration levels and is reported as a range where applicable. Overall, method accuracy was confirmed by comparing the obtained values with the corresponding certified values of the CRMs, demonstrating the reliability of the analytical methods.

### 2.4. Spectral Dataset and Pre-Processing

Spectral pre-processing followed standard chemometric procedures, including combinations of linear detrending, scatter correction (Standard Normal Variate, SNV or Multiplicative Scatter Correction, MSC) [21], and Savitzky–Golay [22] filtering for spectral smoothing or first- or second-order derivative computation (window length: from 3 to 21 points; polynomial order: 2 or 3). Scaling options included mean centring and autoscaling [23]. Identical transformations were applied to calibration and validation datasets.

#### 2.4.1. Spectral Band Selection

Spectral variable reduction was performed using a custom chemometric workflow that combines full-spectrum Partial Least Squares Regression (PLS-R) with interval-based selection based on Variable Importance in Projection (VIP) and Selectivity Ratio (SR) profiles. Following spectral pre-processing, an initial PLS-R model was fitted to the complete wavelength range, and VIP and SR values were computed for each spectral variable (VIP ≥ 1.0 and SR values above the 0.75 quantile). Candidate spectral bands were identified and iteratively combined using stepwise interval PLS (siPLS) to minimise cross-validation error (Root Mean Square Error of Cross-Validation, RMSECV). An optional refinement step was performed using Competitive Adaptive Reweighted Sampling (CARS-PLS) with an exponential decay rate of 0.9, 30 sampling iterations, and a minimum retention threshold of 30 variables.

#### 2.4.2. Model Calibration and Algorithm Testing

**Figure 1:**
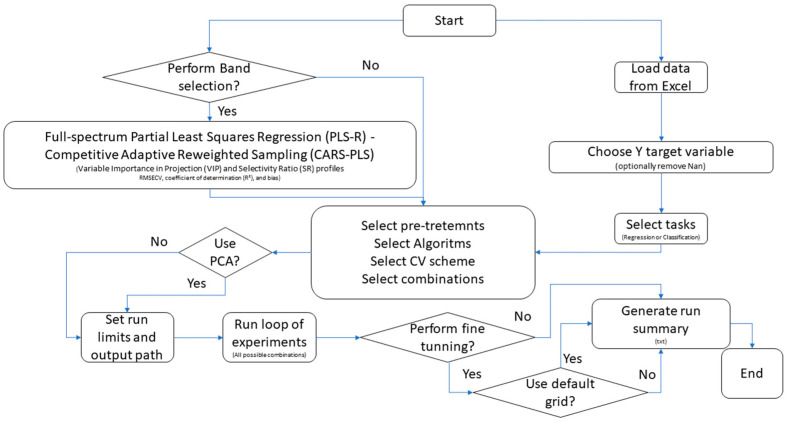
Schematic workflow adopted for the selection of optimal algorithms, pre-processing strategies, and tuning parameters to maximise model performance. Arrows denote the sequential progression of the workflow steps.

Model calibration and evaluation were performed using a customised workflow enabling interactive configuration of spectral pre-processing, algorithm selection, and validation schemes for both regression and classification tasks, depending on the target variable (Figure 1). The following algorithms were evaluated for regression tasks: PLS-R; k-nearest neighbours (k-NN; Euclidean or Manhattan distance); Random Forest (RF; 200–500 trees; maximum depth 10–20); and Support Vector Machines (SVMs; linear, polynomial, radial basis function, and sigmoid kernels). For classification tasks, the corresponding implementations were used (k-NN classifier, RF classifier, and Support Vector Classification, SVC). Model performance was assessed using Venetian-blind cross-validation (5 folds) and bootstrap resampling (100 iterations). For regression, performance was quantified using the coefficient of determination (R^2^), Root Mean Square Error (RMSE) and Mean Absolute Error (MAE). For classification, metrics included overall accuracy, balanced accuracy—BA, defined as the arithmetic mean of class-wise recall (TP rate) across all classes—macro-averaged F1 score, and Matthews Correlation Coefficient (MCC). For classification models, confusion-matrix components—true positives, true negatives, false positives, and false negatives—were also computed and used to derive the following performance indices in Equations (1)–(4) [1].

(1) $$MCC=\frac{TP\cdot TN-FP\cdot FN}{\sqrt{\left(TP+FP)(TP+FN)(TN+FP)(TN+FN\right)}}$$

(2) $$BA=\left(\frac{1}{C}\right)\times \sum_{i=1}^{C}\left[\frac{{TP}_{i}}{\left({TP}_{i}+{FN}_{i}\right)}\right]$$

(3) $${F1}_{i}=2\times \frac{\left({Precision}_{i}\times {Recall}_{i}\right)}{\left({Precision}_{i}+{Recall}_{i}\right)}$$

(4) $${F1}_{macro}=\frac{1}{C}\times \sum_{i=1}^{C}{F1}_{i}$$

where C = number of classes; TPᵢ = true positives for class i; FNᵢ = false negatives for class i; Precisionᵢ = TPᵢ/(TPᵢ + FPᵢ); Recallᵢ = TPᵢ/(TPᵢ + FNᵢ).

The optimal pre-processing–model combinations were further fine-tuned using a grid search approach, which enabled systematic hyperparameter optimisation based on user-defined parameter grids (e.g., the number of latent variables for PLS, the number of neighbours for k-NN, kernel-specific parameters for SVM, and the number of estimators or maximum tree depth for RF) (Table S1).

During model tuning, the optimal hyperparameter configuration was selected based on task-specific performance criteria, namely minimisation of the RMSE for regression models and maximising of the macro-averaged F1 score for classification models.

Two workflows (Figure 1) were compared using identical cross-validation schemes and performance metrics: (*i*) full-spectrum calibration, in which algorithms were applied to the complete spectral range, and (*ii*) band-selected calibration, in which the same algorithms were trained on reduced spectral subsets obtained through siPLS and CARS-PLS selection procedures.

Comparative analyses were conducted to quantify improvements in predictive performance, model robustness, and parsimony attributable to wavelength selection.

### 2.5. Software and Statistical Analysis

All computational analyses were performed in Python 3.12 using the scikit-learn (v1.5), NumPy (v1.26), pandas (v2.2), and matplotlib (v3.9) libraries within the Spyder integrated development environment (IDE) on a Windows 10 operating system. Each analytical run generated dedicated reproducibility files and graphical outputs, as well as complete scripts, hyperparameter grids, and resulting datasets (Excel format), which were systematically archived to ensure full reproducibility of the modelling workflow.

Statistical analyses were conducted using XLSTAT 2023.1.1 (Addinsoft, Paris, France). Data distribution was assessed using the Shapiro–Wilk test prior to parametric testing. Differences among treatment means were evaluated by one-way analysis of variance (ANOVA) according to the experimental design. Statistical significance was declared at *p* < 0.05.

## 3. Results

### 3.1. Effect of Botanical Origin on Wet-Chemistry Quality Traits

Due to their heterogeneous botanical origin, multifloral honeys were not considered a homogeneous group from a compositional standpoint. Although they were included as a functional category for classification purposes, they comprised multiple botanical origins, each represented by a limited number of samples. Consequently, given their lack of botanical uniformity and insufficient sample size per floral source, multifloral honeys were excluded from the ANOVA. They were neither treated as a single uniform group nor analysed as separate individual floral origins.

The results of the one-way ANOVA assessing the effect of botanical origin on the physicochemical composition of honey are reported in Table 1. Significant differences between CH and PF honeys were observed for most of the investigated chemical traits. Compared to PF, CH honeys showed a significantly lower moisture content and also a markedly lower HMF concentration. Electrical conductivity also differed substantially between the two groups, with CH honeys exhibiting significantly higher values, likely consistent with their known mineral composition. Regarding carbohydrate composition, glucose content was significantly lower in CH honeys compared with PF honeys, while fructose content did not differ between categories. Consequently, reducing sugars were significantly lower in CH honeys. CH honeys also exhibited a significantly higher pH than PF honeys.

### 3.2. NIR Performance

#### 3.2.1. NIR Spectra

**Figure 2:**
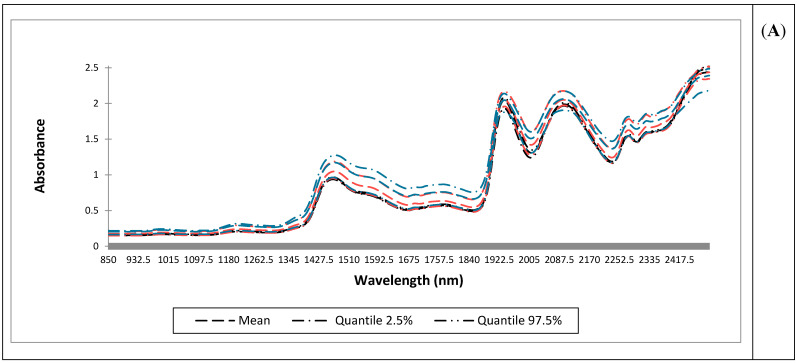

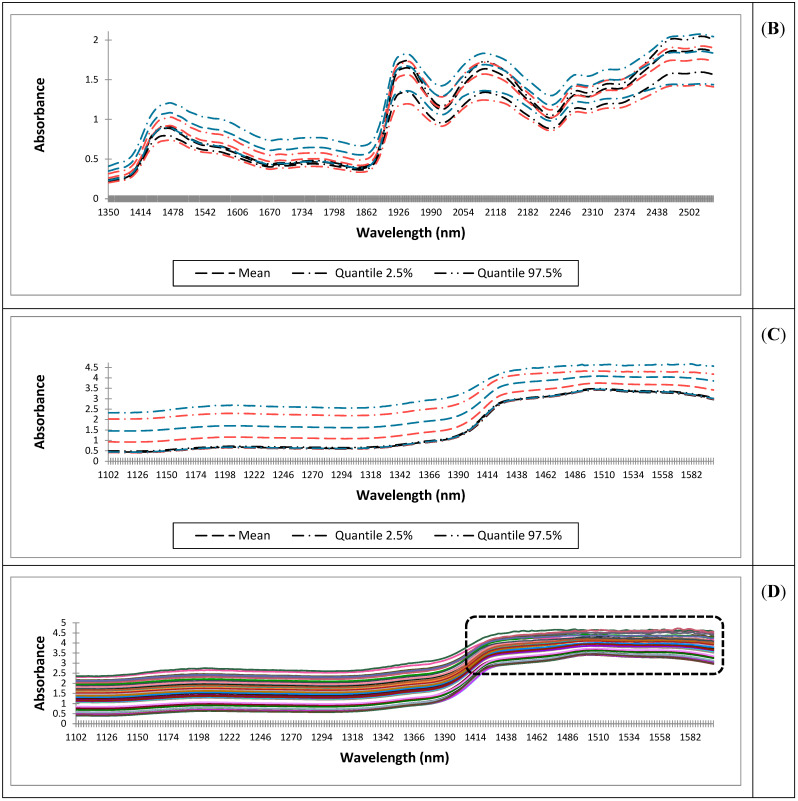
Spectra acquired using the benchtop system (850–2500 nm; 0.5 nm spectral resolution) (Panel (**A**)), the portable wide-range system (Panel (**B**)), and the portable narrow-range system (Panel (**C**)), together with a detailed view of all raw spectra from the portable narrow-range system (Panel (**D**)). Spectra are coloured according to floral group (Panels (**A**–**C**)): Chestnut (CH) in black line, Multifloral in orange line, and Polyfloral (PF) in blue line. Box in Panel (**D**) represents the most noisy region of the raw spectra from the portable narrow-range system.

NIR spectra acquired using the benchtop system (Panel A), the portable wide-range system (Panel B), and the portable narrow-range system (Panel C) are shown in Figure 2. Across instruments, characteristic honey absorption features associated with water and carbohydrate functional groups were evident. For a portable narrow-range system, a detailed view of the raw spectra (Figure 2D) revealed high absorbance values (>2), particularly in the 1400–1600 nm region, where increased noise was observed.

#### 3.2.2. Algorithms Tested Without Spectral Band Selection

The predictive performance of machine learning models for estimating honey physicochemical parameters from NIR spectra is reported in Table 2. Overall, model accuracy varied substantially across parameters and instruments.

Following the analytical workflow reported in Figure 1, multiple regression and classification algorithms were evaluated using full-spectrum data. The best-performing pre-processing and algorithm combinations for each parameter and instrument are reported in Table 2 and Table 3. For the benchtop system, regression models achieved high predictive performance for electrical conductivity (R^2^ = 0.91), glucose (R^2^ = 0.91), reducing sugars (R^2^ = 0.83) and moisture (R^2^ = 0.83), whereas lower predictivity was observed for HMF (R^2^ = 0.45) and diastatic activity (R^2^ = 0.21). SVR, particularly with linear kernels, emerged as the most frequently selected algorithm. Botanical origin classification based on cross-validation achieved high performance (balanced accuracy = 0.90, MCC = 0.78) using an SVC linear model. For the portable wide-range system, predictive performance was generally lower than that obtained with the benchtop instrument, with R^2^ values ranging from 0.09 to 0.85 depending on the parameter. Glucose and electrical conductivity were among the best-predicted variables. The botanical origin classification using a k-NN classifier yielded limited performance (balanced accuracy = 0.50). The k-NN classifier algorithm yielded an overall accuracy of 0.74, but balanced accuracy dropped to 0.50, suggesting class imbalance effects and reduced robustness across minority classes. For the portable narrow-range system, regression models showed variable performance across parameters, with relatively good predictivity for pH (R^2^ = 0.75) and electrical conductivity (R^2^ = 0.71), while HMF and diastatic activity remained poorly predicted. Botanical origin classification using an RF classifier resulted in moderate balanced accuracy (0.62).

#### 3.2.3. Algorithms Tested with Spectral Band Selection

After applying spectral band selection via siPLS and CARS-PLS, model performance was re-evaluated for all instruments (Table 2 and Table 3). For several parameters, wavelength selection resulted in changes in predictive performance compared with full-spectrum models. For the benchtop system, band-selected models led to only marginal improvements in the prediction of pH (R^2^ = 0.77), HMF (R^2^ = 0.49), and reducing sugars (R^2^ = 0.85). Glucose prediction remained consistently high (R^2^ = 0.91), while moisture prediction also maintained good performance (R^2^ = 0.83). Classification of botanical origin achieved a balanced accuracy of 0.55 using a k-NN classifier. For the portable wide-range system, modest improvements were observed for moisture (R^2^ = 0.75) and pH (R^2^ = 0.73). Glucose prediction maintained good predictive performance (R^2^ = 0.85), whereas classification performance remained limited (balanced accuracy = 0.50). For the portable narrow-range system, band selection marginally improved the prediction of selected parameters such as electrical conductivity (R^2^ = 0.75) and moisture (R^2^ = 0.73), while classification performance reached a balanced accuracy of 0.57 using RF models.

## 4. Discussion

### 4.1. Interpretation of the Effect of Botanical Origin on Wet-Chemistry Quality Traits

Significant differences between CH and PF honeys were observed for most of the analysed chemical variables (Table 1), but they all complied with the limits established by Directive 2001/110/EC. Considering the sugar composition in CH samples, they exhibited a higher fructose content and a lower glucose concentration compared to PF, according to the literature [24]. Honey crystallisation is a naturally occurring process that is mainly governed by sugar composition and water availability. Therefore, to ensure sample homogeneity, honeys were heated at 30 °C for 30 min before analysis. The higher fructose-to-glucose ratio (1.89 and 1.27 for CH and PF, respectively) in CH samples may explain their greater fluidity and clarity, whereas PF samples, characterised by a higher glucose content, exhibited a greater tendency to crystallise. Honeys with a high glucose content (28–30 g/100 g) or a fructose-to-glucose ratio below 1.14 are reported to crystallise more rapidly [25]. From a quality standpoint, crystallisation is generally considered undesirable, as it leads to the formation of coarse crystals that negatively affect texture and visual appearance, thereby reducing consumer acceptance and complicating handling during processing and packaging [26,27]. From an NIR perspective, differences in crystallisation state are expected to strongly influence spectral responses through changes in light scattering and water–sugar interactions, potentially contributing to the divergent spectral patterns observed between CH and PF samples [25].

The differences observed between CH and PF samples can be interpreted in light of the strong influence of botanical origin on honey composition, crystallisation behaviour, colour, and spectral response. In line with the descriptive framework provided by [24], CH samples showed higher fructose content and higher F/G ratios, which are known to favour a more fluid and stable liquid state, whereas PF samples, characterised by relatively higher glucose levels, exhibited a greater tendency to crystallise. This behaviour is consistent with the crystallisation thresholds reported by [25].

From a spectroscopic perspective, our NIR results are consistent with those of [25], who demonstrated that crystallisation state and botanical origin strongly modulate NIR spectral patterns through changes in light scattering and water–sugar interactions. Accordingly, the divergent spectral responses observed between CH and PF samples are likely attributable not only to compositional differences but also to differences in physical state, with crystallised matrices exhibiting stronger scattering effects and more pronounced water-structure rearrangements. This confirms that NIR spectral variability may reflect physical restructuring rather than chemical changes alone. Although heating can dissolve sugar crystals and delay crystallisation, it does not eliminate honey’s intrinsic tendency to recrystallise, which is primarily governed by sugar composition [26,27]. Overall, our findings confirm that botanical origin strongly shapes honey crystallisation behaviour and NIR spectral signatures and should therefore be considered when interpreting spectroscopic data across honeys differing in botanical origin or physical state.

The honey samples tested in this study presented a high degree of freshness, as indicated by their high diastatic activity (approximately 29 Schade Units on average) and low HMF content (5.9 mg/kg) if compared to literature [18] and to the limits established by the EU legislation. The two floral origins showed significant differences in pH and electrical conductivity. These parameters are closely related to the concentration of mineral salts, organic acids, phenolic compounds, and proteins [28]. CH honey is particularly rich in polyphenols [29], which appear to enhance the thermal stability of the enzymes present in honey and the electrical conductivity [30]. Differences in water-related parameters are expected to strongly influence spectral responses through changes in water–solute interactions, potentially contributing to the observed divergent spectral patterns. Thermal treatment has been shown to significantly affect honey pH, with increasing temperatures inducing a progressive decrease in pH and a concomitant increase in free acidity, reflecting heat-induced chemical transformations of sugars and related compounds [27], and differences in acidity may contribute indirectly to variations in water–sugar interactions and, consequently, to spectral responses observed among honeys with different botanical origins and crystallisation states [25].

### 4.2. Interpretation of the NIR Spectra

NIR spectra acquired using the benchtop instrument (Panel A) and the portable wide- (Panel B), and narrow-range systems (Panels C, D) are shown in Figure 2. For all instruments the shapes of the spectra resemble those reported by [31,32], confirming the presence of four dominant absorbance bands: the regions from 1420 to 1470 nm and from 1900 to 1940 nm, both associated with O–H, C–H, and C–H_2_ deformations (which can be attributed to the presence of carbohydrates and water), and the region from 2050 to 2150 nm, which corresponds to C–H combination bands, typically of carbohydrates, as reported by [33].

Regarding the three classes, the mean absorbance was consistently higher for PF samples, while CH samples displayed the lowest values, and the pooled multifloral class presented intermediate values across all instruments. These differences are likely related to variations in sugar composition, water–sugar interactions, and crystallisation state, which are known to strongly influence NIR spectral responses. The pooled multifloral class is consistent with its broader compositional variability and mixed botanical origin, as reflected in the spectral information obtained from all instruments. Accordingly, heterogeneous or mixed-origin honeys displayed intermediate absorbance values due to combined absorption–scattering behaviour, as commonly observed in complex food matrices [5,34]. However, thermal processing can temporarily reduce scattering by dissolving sugar crystals without altering the intrinsic compositional drivers of crystallisation [26].

In Panel D of Figure 2, a detailed representation of all raw spectra acquired with the portable narrow-spectrum system is reported. The plot indicates that CH samples characterised by lower absorbance values did not exhibit pronounced noise, whereas PF samples, showing higher absorbance levels, displayed more evident noise effects. Absorbance values exceeding 2—and in some cases reaching values above 4.5—are likely attributable to an excessive optical pathlength, possibly caused by the use of vials with excessive thickness, which limited the amount of light reaching the detector. Given that PF samples exhibited the highest absorbance values, the presence of sugar crystals may have further contributed to the observed increase in spectral noise through enhanced light scattering.

The following sections present the comparative results obtained with the tested algorithms. Regardless of the algorithm or spectral range, spectral pre-processing had a marked impact on model performance. In NIR honey analysis, pre-processing is essential to correct scattering effects, baseline shifts, and instrumental noise. Techniques such as SNV and MSC reduce scatter-related variability, while detrending corrects baseline drift. Savitzky–Golay filtering enables smoothing and derivative computation, improving the resolution of overlapping bands. The choice and combination of pre-processing steps strongly influence model robustness and predictive accuracy, making pre-processing a key factor in calibration development and transferability [15,35]. Consequently, pre-processing is not a trivial step but a key determinant of calibration accuracy and transferability in honey spectroscopy studies. However, the optimal pre-processing strategy remains an open and debated issue. NIR signals inherently contain both chemical information and unwanted variability (e.g., noise, background effects), which can only be partially corrected through pre-processing [36]. As a result, no standardised procedure exists, and pre-processing selection often relies on a trial-and-error approach, introducing a degree of subjectivity [37].

Common strategies include first- and second-derivative transformations to correct baseline shifts and enhance peak separation, and SNV to mitigate multiplicative effects related to scattering, particle size, and sample presentation [38]. Given the large number of possible pre-processing combinations, the present study did not aim to isolate the effect of individual methods. Instead, the reported results reflect the best-performing combinations of pre-processing and algorithms, selected by maximising R^2^ for regression and balanced accuracy for classification.

#### 4.2.1. Comparative Results of Tested Algorithms on Regression and Classification Tasks Without Band Selection

The best-performing combinations of pre-processing and algorithms for each parameter and instrument are reported in Table 2 and Table 3. In the benchtop instrument, regression task performance varied considerably across chemical parameters. Electrical conductivity and glucose showed the highest predictive performance, with R^2^ values of 0.91; other parameters, such as moisture and reducing sugars, also showed promising regression performance (R^2^ = 0.83). The metrics for electrical conductivity and glucose were consistent with the results previously reported by [39]; in contrast, the humidity metrics were lower than those reported by the same authors, with an R^2^ value of 0.97.

For reducing sugars, the lower R^2^ value was consistent with the findings reported by [39] and may be attributed to the higher variability observed (as reflected by the RMSE and MAE values), together with the analytical procedure [40]. Specifically, the reference solutions used for calibration curve construction contained known concentrations of glucose, fructose, and sucrose standards but did not account for other reducing sugars present in honey [41]. The presence of these additional sugars may have introduced greater variability, thereby reducing model accuracy and predictive performance.

Conversely, HMF and diastatic activity yielded substantially lower R^2^ values, indicating that these parameters are challenging to model using NIR spectral data. This limitation is consistent with their low concentration levels and functional nature, as well as with the higher analytical uncertainty of the corresponding reference methods, which collectively constrain NIR prediction performance, as previously reported for heat-related quality indicators in honey [25,27,39]. These findings are also consistent with those reported by [42], who observed particularly poor predictability for HMF and diastatic activity; the performance metrics obtained are also consistent with those reported by [43]. Considering diastatic activity, the limited predictive capacity can be attributed to the naturally low concentrations of this enzyme in honey, which result in weak, overlapping NIR absorption features that are easily masked by the broad overtone and combination bands of water and carbohydrates, which dominate the spectrum [25,27,34,40].

For botanical origin classification, the linear SVC achieved a balanced accuracy of 0.90 (F1 = 0.88, MCC = 0.78), indicating robust, well-balanced discrimination across floral classes under bootstrap cross-validation. The reported balanced accuracy and MCC support the classification’s reliability in the presence of heterogeneous spectral variability, consistent with previous NIR-based classification and screening studies in complex food matrices [40] and with methodological recommendations for scatter-dominated spectra [5].

For the portable wide-range system, as with the previous instrument, regression performance varied across the chemical parameters. For moisture, the obtained R^2^ value (0.66) was lower than that of the benchtop NIR, while RMSE and MAE were higher, indicating a reduced ability of the portable systems to accurately capture data variance, as previously reported for NIR measurements under lower spectral resolution and signal-to-noise conditions [34,40].

HMF and diastatic activity showed even lower R^2^ values (0.32 and 0.09, respectively), confirming that these quality markers remain challenging to model using NIR spectroscopy due to their low concentration, functional nature, and limited spectral specificity [25,27]. Electrical conductivity was also less effectively predicted (R^2^ = 0.68), consistent with the indirect relationship between this parameter and NIR-active constituents, which are more sensitive to instrumental noise and matrix effects [5].

In contrast, glucose exhibited high predictive quality (R^2^ = 0.85), likely due to the presence of strong carbohydrate-related absorption bands (1420–1470 nm, 1900–1940 nm (O–H, C–H, and C–H_2_ deformations), and 2050–2150 nm (C–H combination bands)) within the spectral range covered by the instrument, as widely reported for honey NIR spectra [25,34].

For the classification task, the k-NN classifier model developed for botanical origin discrimination achieved a balanced accuracy of 0.50, meaning weak discrimination of botanical origin, in line with previous observations on the limitations of portable NIR systems for complex multi-class classification problems [40].

For the portable narrow-range system, among the evaluated parameters, electrical conductivity, glucose, and reducing sugars showed the highest predictive performance, achieving R^2^ values of 0.71, 0.86, and 0.70, respectively, which indicate moderate predictive accuracy compared to the benchtop system. This pattern is consistent with the fact that these parameters are either directly related to carbohydrate composition or indirectly influenced by water–sugar interactions, which dominate NIR spectral responses in honey [25,34]. These results are consistent with the instrument’s extremely limited spectral acquisition range (1102–1600 nm). Within this range, only the first overtone and combination bands associated with O–H and C–H (including C–H_2_) vibrations can be detected, which explains why parameters linked to water and sugars were predicted with greater accuracy. As reported in previous studies, this restricted range primarily captures information related to bulk constituents such as water and sugars, while limiting sensitivity to minor or functionally defined parameters [5,40]. Consequently, parameters linked to water and carbohydrate content were predicted with comparatively better accuracy, whereas properties less directly associated with strong NIR absorption features remained more challenging to model using portable, narrow-range instruments.

For the classification task, the RF model developed for botanical origin discrimination achieved a balanced accuracy of 0.62, indicating overall moderate classification performance among botanical classes, but class separation remained limited, particularly in the presence of high intra-class heterogeneity and overlapping spectral features. Similar moderate classification outcomes have been reported in NIR-based studies dealing with complex food matrices, where variability associated with botanical origin, crystallisation state, and water–sugar interactions can mask subtle compositional differences [25,34]. Across the classification models tested, clear differences in performance highlight the combined effects of algorithm choice, spectral quality, and experimental conditions. The linear SVC consistently provided the most reliable discrimination, achieving a balanced accuracy of 0.90 (for the benchtop system), indicating good classification performance and suggesting that the spectral features relevant for botanical origin discrimination were not substantially altered by the increased temperature. In contrast, the RF model showed only moderate discriminative ability (balanced accuracy = 0.62), likely reflecting sensitivity to noise, redundancy, and limited spectral information. The k-NN classifier performed weakest (balanced accuracy ≈ 0.50), consistent with its reliance on local distance measures and its susceptibility to overlapping class distributions. Overall, these results indicate that, under conditions of limited spectral range and strong matrix effects, linear classifiers tend to be more stable than ensemble- or distance-based methods, while classification performance remains constrained, supporting the use of portable NIR systems primarily for screening rather than confirmatory botanical authentication [5,25,40].

#### 4.2.2. Comparative Results of Tested Algorithms on Regression and Classification Tasks After Band Selection

Across all systems, the metrics obtained after band selection varied depending on the parameter, with only limited improvements observed, particularly for moisture, electrical conductivity, and pH. The effectiveness of wavelength selection for moisture prediction can be attributed to the strong and well-defined NIR absorption bands of water, especially the prominent O–H absorptions around 1400–1450 nm, corresponding to the first overtone of O–H stretching, and around 1900–1950 nm, associated with O–H stretching and bending combination bands [33]. Although all systems were capable of detecting at least one of these features, the higher spectral resolution and signal-to-noise ratio of the benchtop instrument likely explained its superior performance. For portable systems, wavelength selection may enhance the signal-to-noise ratio by removing non-informative spectral regions, resulting in modest improvements in model robustness and predictive accuracy.

Similar considerations apply to pH and electrical conductivity; however, since these parameters were not directly associated with specific NIR-active constituents, their prediction was more sensitive to instrumental noise and matrix effects [5]. Accordingly, the best results were obtained with the benchtop and the portable wide-range system, likely due to their broader spectral coverage and improved signal-to-noise ratio.

For carbohydrate composition, all systems achieved good predictive performance (R^2^ values up to 0.85), comparable to models developed without band selection. Sugar molecules exhibited characteristic spectral features arising from vibrational overtones and combination bands, primarily associated with O–H and C–H functional groups [15]. The benchtop instrument consistently achieved the best performance, both with and without band selection. This suggests that, because spectral information related to carbohydrate composition is distributed across a wide spectral range, predictive performance depends more on spectral resolution and signal-to-noise ratio than on the selection of specific wavelengths.

To support this interpretation, the portable systems, characterised by lower spectral resolution and less favourable signal-to-noise ratios, consistently showed lower predictive accuracy for glucose and reducing sugars. For HMF, predictive performance was consistently poor across all systems. This may be attributed to the limited number of samples and the narrow concentration range included in this study. Moreover, all samples exhibited relatively low and homogeneous HMF concentrations (mean values of 5.87 mg/kg for CH and 12.7 mg/kg for PF), well below the regulatory limit. This limited variability likely constrained model calibration, as robust prediction of HMF typically requires a larger number of samples covering a wider concentration range [43]. The spectral region between 2063 and 2352 nm is associated with combination bands of C–O, C–H, and O–H stretching vibrations, which correspond to the main functional groups of HMF [44]. This region is accessible only to the benchtop and the portable wide-range system, whereas the portable narrow-spectrum system is limited to the 1000–1250 nm range, corresponding mainly to second overtone C–H stretching. Also in this case, the higher spectral resolution and signal-to-noise ratio of the benchtop instrument likely explained its superior performance. Diastatic activity showed the lowest predictability, consistent with previous findings [43]. Band selection did not improve predictive performance for any systems, likely due to the low concentration of this enzyme in honey, which produced weak and highly overlapping NIR absorption features. These subtle signals were masked by the dominant overtone and combination bands of major honey constituents, such as water and carbohydrates [25,27,34,40]. For botanical origin classification, band selection did not improve model performance. The best results after band selection were obtained with the benchtop and portable narrow-spectrum systems, with balanced accuracy values of 0.55 and 0.57 and MCC values of 0.65 and 0.60, respectively; however, these were lower than those achieved using the full spectrum. This may be explained by the fact that these two systems covered the key spectral region for botanical discrimination (1585–1710 nm), as reported by [45] and confirmed by the most informative wavelengths reported in Table S2. However, the higher spectral resolution and signal-to-noise ratio of the benchtop instrument enabled more effective extraction of discriminative spectral information, resulting in better overall performance.

The authors recognise that external validation using an independent dataset would represent the most rigorous strategy to prevent overly optimistic estimates of model performance. Nonetheless, the limited sample size in the present study did not allow a statistically robust separation of calibration and test sets without substantially reducing model reliability. Furthermore, the honey samples were sourced from the commercial market and originated from heterogeneous and randomly distributed production contexts, minimising the likelihood of systematic clustering related to geographical origin or processing conditions. Under these circumstances, the adoption of repeated cross-validation with random sample allocation was considered an acceptable compromise to approximate external validation. Nevertheless, the absence of an independent validation set remains a limitation of the study. Therefore, the authors report the present findings with a cautious, moderate level of confidence, acknowledging the need for confirmation using independent datasets and further external validation studies to substantiate the generalizability and robustness of the proposed models.

### 4.3. Overall Discussion and Suggestions

Notably, the three systems evaluated differ not only in spectral range but also in spectral resolution, acquisition geometry, and wavelength-selection principle. The observed differences in calibration performance are therefore attributable to the combined effect of these instrumental factors, rather than to any single variable. In particular, the superior performance of the benchtop system may reflect the advantages of its broader spectral coverage, higher spectral resolution, and greater signal stability, which are inherent to scanning monochromator-based benchtop systems operating under controlled laboratory conditions. The analytical performance observed for the specific instruments evaluated in the present study cannot be generalised to the broader landscape of available NIR instrumentation, as other systems—including both benchtop and portable systems—may yield substantially different calibration outcomes depending on their design specifications, optical configuration, and operating conditions [46,47]. Accordingly, the results reported here should be interpreted as instrument-specific rather than as a general statement about the relative capabilities of benchtop versus portable NIR technology. Despite their numerous advantages in terms of compact size, flexibility, and affordability, miniaturised NIR spectrometers may still lag behind benchtop counterparts in certain applications due to performance-limiting factors such as narrower spectral range, lower spectral resolution, and reduced signal-to-noise ratio [47]. Nevertheless, even though spectroscopy techniques are the most commonly used in the area of honey quality assessment, the realistic feasibility of the predictive performance of portable instruments can be improved based on spectral resolution and recording configuration improvements; however in many applications, portable instruments have demonstrated great potential and affordable capacity for: (i) identifying adulterants such as glucose, molasses, nectar [48]; (ii) rapid screening for checking honey adulteration [49]; (iii) testing moisture and water activity with relative strong predictive performance [50]; (iv) the development of models with levels of prediction similar to those of benchtop system in tracing honey botanical and geographical origins [17]. The instruments evaluated in this study do not cover the full range of market availability; rather, they represent three representative configurations—one high-end benchtop scanning monochromator and two portable systems operating on different technological principles and measurement modes—selected to reflect practical deployment scenarios in honey analysis. As established in comparative food analysis studies [51], portable NIR systems can achieve calibration accuracy comparable to benchtop FT-NIR instruments, particularly when appropriate chemometric pre-processing strategies are applied. The present work, therefore, documents the predominant challenges encountered in practical honey analysis across these configurations, rather than making a general claim about the superiority of benchtop technology. The proposed systems offer a rapid, non-destructive, and cost-effective approach for routine analysis, enabling high-throughput screening with minimal sample preparation while supporting both laboratory-based and in-field applications to assess concentrations within the calibration range, which covers the natural variability of Italian honey products. However, these considerations highlight that the choice of instrument should be driven by the intended deployment scenario—laboratory-based precision analysis versus field or in-line applications—rather than by a categorical assumption of technological superiority.

## 5. Conclusions

This study comparatively assessed the analytical performance of one benchtop and two portable NIR systems, used for honey compositional prediction and botanical origin discrimination using a unified chemometric framework. The benchtop system consistently provided higher predictive accuracy and more reliable classification, particularly for parameters associated with carbohydrate and mineral content, confirming the relevance of spectral range and resolution for quantitative robustness. Portable systems achieved moderate accuracy for major compositional traits, supporting their applicability as rapid screening tools, but showed limitations for low-concentration or functionally defined parameters. Variable selection strategies yielded only marginal improvements, indicating that spectral quality plays a greater role than wavelength reduction in this matrix. Overall, the results clearly differentiate the operational domains of the tested systems, supporting portable NIR systems for preliminary field screening and benchtop platforms for laboratory-grade quantification and authentication, and highlight the need for instrument-specific calibration models to ensure reliability and transferability.

## Figures and Tables

**Table 1:** Effect of the botanical origin of the sampled honey (*n* = 30 CH + 30 PF) on the physicochemical parameters.

Variable/Category	CH	PF	*p*-Value	SEM
Moisture (%)	17.1	17.7	0.020	0.18
HMF (mg/kg)	5.87	12.7	0.001	1.01
Diastatic activity (Schade unit)	29.3	26.9	0.215	1.39
Electrical conductivity (mS/cm)	1.92	0.79	0.001	0.06
Glucose (%)	20.4	30.2	0.001	0.46
Fructose (%)	38.5	38.4	0.894	0.51
Reducing sugars (%)	58.9	68.6	0.001	0.72
pH	5.11	4.10	0.001	0.06

CH = chestnut; PF = polyfloral; HMF = Hydroxymethylfurfural; SEM = standard error of the mean. Statistical significance was declared at *p* < 0.05.

**Table 2:** Performance of machine learning models developed for the prediction of physicochemical parameters in honey samples based on NIR spectral data. The table reports the selected models, spectral pre-processing strategies, the number of latent variables (for PLS) or principal components (PCs) retained after principal component analysis (PCA), the cross-validation (CV) scheme, and model performance metrics. Regression performance is expressed as the coefficient of determination (R^2^), root mean square error (RMSE) and mean absolute error (MAE). Results are reported for whole spectra or after spectral band selection for: the benchtop instrument (FOSS DS-2500, 850–2500 nm; 0.5 nm resolution), a portable wide-range system (NeoSpectra™ spectrometer, 1350–2500 nm; 16 nm resolution), and a portable narrow-range system (AlbaNIT spectrometer, 1102–1600 nm; 2 nm resolution). Spectral bands were identified and iteratively combined using a stepwise interval PLS (siPLS) and an optional refinement step was performed using Competitive Adaptive Re-weighted Sampling (CARS-PLS).

	Model	Pre-Processing	PC	CV Scheme	R^2^	RMSE	MAE
Full spectrum							
Benchtop instrument							
Moisture	SVM (linear)	MSC + SG (7) 1st derivative + mean centring + scaling	10	VB5	0.83	0.39	0.31
HMF	RF	Linear detrending + SG (15) 1st derivative + mean centring	5	VB5	0.45	11.20	6.97
Diastatic activity	RF	SG (9) smoothing + mean centring + scaling	2	VB5	0.21	6.59	5.55
Electrical conductivity	SVM (linear)	SNV + linear detrending + SG (9) smoothing + mean centring + scaling	10	VB5	0.91	0.19	0.15
Glucose	SVM (linear)	SNV + linear detrending + SG (15) smoothing + scaling	8	VB5	0.91	1.50	1.15
Fructose	SVM (linear)	SG (9) smoothing + mean centring + scaling	8	VB5	0.59	1.68	1.26
Reducing sugars	SVM (linear)	MSC + SG (7) smoothing + mean centring + scaling	9	B100	0.83	2.24	1.69
pH	SVM (linear)	Linear detrending + SG (13) smoothing + scaling	10	VB5	0.67	0.31	0.23
Portable wide-range system							
Moisture	PLS	SNV + linear detrending + SG (7) 1st derivative	5	VB5	0.66	0.56	0.44
HMF	RF	MSC + SG (11) 2nd derivative	9	VB5	0.32	12.00	7.90
Diastatic activity	RF	SNV + linear detrending + SG (11) 1st derivative	5	VB5	0.09	7.05	5.44
Electrical conductivity	RF	SNV + SG (13) smoothing	9	VB5	0.68	0.35	0.27
Glucose	PLS	SG (13) 1st derivative	5	VB5	0.85	2.07	1.68
Fructose	RF	SG (13) smoothing	9	VB5	0.43	1.99	1.53
Reducing sugars	PLS	SNV + SG (15) 1st derivative	5	VB5	0.67	3.44	2.72
pH	RF	MSC + SG (13) smoothing	9	VB5	0.65	0.34	0.26
Portable narrow-range system							
Moisture	SVM (linear)	Linear detrending + SG (11) 1st derivative + mean centring + scaling	7	VB5	0.68	0.53	0.40
HMF	RF	SG (19) 1st derivative + mean centring	4	VB5	0.46	11.40	7.06
Diastatic activity	RF	Linear detrending + SG (1) smoothing + mean centring + scaling	3	VB5	0.18	6.61	5.30
Electrical conductivity	SVM (linear)	MSC + SG (15) smoothing + mean centring + scaling	7	B100	0.71	0.34	0.26
Glucose	RF	MSC + SG (17) 2nd derivative + mean centring + scaling	7	VB5	0.86	1.89	1.35
Fructose	RF	SNV + SG (15) 1st derivative + scaling	10	VB5	0.28	2.21	1.80
Reducing sugars	SVM (linear)	SG (11) 1st derivative + scaling	7	VB5	0.70	3.29	2.55
pH	RF	SNV + SG (13) 2nd derivative + mean centring + scaling	1	VB5	0.75	0.30	0.24
Spectral band selection							
Benchtop instrument							
Moisture	SVM (linear)	SNV + linear detrending + SG (7) smoothing + mean centring + scaling	7	VB5	0.83	0.37	0.29
HMF	SVM (linear)	Linear detrending + SG (7) 1st derivative + mean centring + scaling	10	B100	0.49	10.80	6.85
Diastatic activity	SVM (polynomial)	SG (7) 2nd derivative + mean centring + scaling	2	VB5	0.05	7.18	5.84
Electrical conductivity	SVM (linear)	Linear detrending + SG (5) 1st derivative + mean centring + scaling	9	VB5	0.84	0.26	0.20
Glucose	RF	MSC + SG (7) 2nd derivative + scaling	6	B100	0.91	1.50	1.18
Fructose	RF	SNV + SG (3) 1st derivative + scaling	8	VB5	0.64	1.62	1.21
Reducing sugars	SVM (linear)	Linear detrending + SG (7) 1st derivative + mean centring + scaling	10	B100	0.85	2.30	1.70
pH	RF	MSC + SG (7) 1st derivative + mean centring + scaling	10	VB5	0.77	0.28	0.21
Portable wide-range system							
Moisture	PLS	SG (11) smoothing	5	VB5	0.75	0.46	0.37
HMF	PLS	no Savitzky–Golay filtering	5	VB5	0.15	14.00	8.07
Diastatic activity	SVM (sigmoid)	SG (3) smoothing	1	VB5	0.07	7.09	5.80
Electrical conductivity	k-NN	SNV + linear detrending + SG (3) 2nd derivative + scaling	7	VB5	0.69	0.35	0.26
Glucose	SVM (sigmoid)	SNV + SG (13) smoothing	1	VB5	0.85	2.03	1.53
Fructose	RF	Linear detrending + no Savitzky–Golay filtering	7	VB5	0.49	1.91	1.57
Reducing sugars	k-NN	SG (7) 2nd derivative	1	VB5	0.64	3.06	2.50
pH	PLS	SG (11) smoothing	5	VB5	0.73	0.36	0.26
Portable narrow-range system							
Moisture	SVM (linear)	SG (9) smoothing + mean centring + scaling	5	VB5	0.73	0.48	0.39
HMF	RF	SNV + linear detrending + SG(11)2nd derivative + mean centring + scaling	1	VB5	0.33	11.20	8.01
Diastatic activity	SVM (polynomial)	Savitzky–Golay filtering + mean centring	4	VB5	0.01	7.26	5.94
Electrical conductivity	SVM (linear)	SG (21) smoothing + scaling	4	VB5	0.75	0.32	0.23
Glucose	SVM (linear)	SG (5) smoothing + mean centring + scaling	9	VB5	0.85	2.02	1.50
Fructose	RF	MSC + SG (5) smoothing	7	VB5	0.16	2.39	1.86
Reducing sugars	k-NN	SG (9) 2nd derivative + mean centring + scaling	8	VB5	0.69	3.19	2.42
pH	SVM (RBF)	SNV + linear detrending + SG (9) smoothing + mean centring + scaling	3	VB5	0.63	0.36	0.25

Abbreviations: HMF, hydroxymethylfurfural; SVM, support vector machine; RF, random forest; MSC, multiplicative scatter correction; SNV, standard normal variate; PC, principal component; R^2^, coefficient of determination; RMSE, root mean square error; MAE, mean absolute error; VB5, Venetian blind five-fold cross-validation; B100, bootstrap resampling with 100 replicates. Spectral pre-processing: spectral variables were pre-processed prior to model calibration. Pre-treatments included scatter correction (MSC or SNV), Savitzky–Golay filtering (smoothing or derivative transformation), and scaling methods (mean centring or autoscaling). The Savitzky–Golay filter was applied to the spectral data as a polynomial smoothing or derivative filter using various window sizes, reported in brackets.

**Table 3:** Performance of machine learning models developed for the prediction of botanical origin in honey samples based on NIR spectral data. The table reports the selected models, spectral pre-processing strategies, the number of latent variables (for PLS) or number of principal components (PCs) retained after principal component analysis (PCA), cross-validation scheme (CV), and model performance metrics. Classification performance is reported as accuracy, balanced accuracy, F1-score, and Matthews correlation coefficient (MCC). Results are reported for whole spectra or after spectral band selection for: the benchtop instrument (FOSS DS-2500, 850–2500 nm; 0.5 nm resolution), the portable wide-range system (NeoSpectra™ spectrometer, 1350–2500 nm; 16 nm resolution), and the portable narrow-range system (AlbaNIT spectrometer, 1102–1600 nm; 2 nm resolution). Spectral bands were identified and iteratively combined using a stepwise interval PLS (siPLS) and an optional refinement step was performed using Competitive Adaptive Re-weighted Sampling (CARS-PLS).

	Model	Pre-Processing	PC	CV Scheme	Accuracy	BA	F1-Score	MCC
Full spectrum								
Benchtop instrument								
Botanical origin	SVM (linear)	SNV + SG (9) 1st derivative + mean centring + scaling	4	B100	0.91	0.90	0.88	0.78
Portable wide-range system								
Botanical origin	k-NN	MSC + SG (11) 2nd derivative	8	VB5	0.74	0.50	0.44	0.62
Portable narrow-range system								
Botanical origin	RF	SNV + SG (5) smoothing	9	VB5	0.78	0.62	0.55	0.70
Spectral band selection								
Benchtop instrument								
Botanical origin	k-NN	SG (5) smoothing + mean centring	4	B100	0.76	0.55	0.49	0.65
Portable wide-range system								
Botanical origin	RF	SG (3) smoothing	9	B100	0.69	0.50	0.44	0.54
Portable narrow-range system								
Botanical origin	RF	SG (3) 1st derivative	5	B100	0.72	0.57	0.52	0.60

Abbreviations: SVM, support vector machine; RF, random forest; MSC, multiplicative scatter correction; SNV, standard normal variate; PC, principal component; CV scheme: VB5, Venetian blind five-fold cross-validation; B100, bootstrap resampling with 100 replicates; BA, balanced accuracy. Spectral pre-processing: spectral variables were pre-processed prior to model calibration. Pre-treatments included scatter correction (MSC or SNV), Savitzky–Golay filtering (smoothing or derivative transformation), and scaling methods (mean centring or autoscaling). The Savitzky–Golay filter was applied to the spectral data as a polynomial smoothing or derivative filter using various window sizes, reported in brackets.

## Data Availability

The data presented in this study are openly available at Research Data Unipd, the open-source repository of databases at the University of Padova (DOI: https://doi.org/10.25430/researchdata.cab.unipd.it.00001770) at: https://researchdata.cab.unipd.it/1770/ (accessed on 31 March 2026).

## Supplementary Materials

The following supporting information can be downloaded at: https://www.mdpi.com/article/10.3390/s26092796/s1, Table S1: Default grid used for systematic hyperparameter optimisation based on user-defined parameter grids. Table S2: Selected wavelengths for each device were identified as the most informative by the spectral band selection approach.
