# Supplementary material for: Rapid Assessment of Italian Honey Chemical Composition and Botanical Origin Using NIR Spectroscopy Coupled with Chemometric Analysis
Source: Sensors (Basel). 2026 Apr 30;26(9):2796. doi: 10.3390/s26092796 (PMC13165706; doi:10.3390/s26092796)
Supplement: Supplementary file 1 [file sensors-26-02796-s001.zip › sensors-4226307-supplementary.pdf]

Table S1. Default grid used for systematic hyperparameter optimisation based on user-defined parameter grids.

For Partial Least Squares Regression (PLSR), the number of latent variables was varied in the range 2–10 with even spacing.
For k-Nearest Neighbours (k-NN), the grid included<br>• number of neighbours = {3, 5, 7, 9, 11},<br>• weights = {'uniform', 'distance'},<br>• metric = {'euclidean', 'manhattan'}.
For Random Forest models (both regression and classification), parameter combinations were drawn from<br>• number of estimators = {200, 500},<br>• max_depth = {None, 10, 20},<br>• max_features = {'sqrt', 0.8, 1.0}.
For Support Vector Machines (SVR or SVC), the grid explored<br>• C = {0.1, 1, 10},<br>• gamma = {'scale', 'auto'},<br>• degree = {2, 3},<br>• tested across kernels (linear, polynomial, radial-basis, and sigmoid).

Table S2. Selected wavelengths for each device were identified as the most informative by the spectral band selection approach.

Parameter	FOSS DS-2500 (850 - 2500 nm, resolution: 0.5 nm)	Portable wide-range device (1350 - 2500 nm, resolution: 16 nm)	Portable narrow-range device (1102 - 1600 nm, resolution: 2nm)
Moisture	940.5-953.5 nm, 965 nm, 978.5-981.5 nm, 991.5-995.5 nm, 1121-1123 nm, 1302.5-1312.5 nm, 1485.5-1488 nm, 1496-1501 nm, 2236.5-2240 nm, 2242-2245.5 nm, 2247.5-2251.5 nm, 2253.5-2255.5 nm, 2258-2260.5 nm 2263-2265.5 nm	1802-1818 nm, 1916-1924 nm, 2356-2386 nm	1142-1170 nm, 1194-1214 nm, 1284-1316 nm, 1394-1442 nm, 1466-1526 nm
HMF	894-902 nm, 943.5-963.5 nm, 1304-1315 nm, 1760.5-1771.5 nm, 2253.5-2284 nm	1444-1484 nm, 2356-2366 nm	1412-1430 nm, 1468-1472 nm, 1482 nm, 1488-1492 nm, 1498-1528 nm
Diastatic activity	1311.5-1324 nm	1712-1730 nm	1444-1452 nm
Electrical conductivity	1006-1014 nm, 1018-1037.5 nm, 1045.5-1056 nm, 1062.5-1066.5 nm, 1071.5-1075.5 nm, 1462.5-1470.5 nm, 1472.5-1473.5 nm, 1690.5-1695 nm, 1697-1704.5 nm, 1706-1709.5 nm	2310-2332 nm, 2374-2452 nm	1392-1396 nm, 1406 nm, 1508-1520 nm, 1524-1544 nm, 1548-1574 nm
Glucose	1014.5-1023.5 nm, 1219.5-1220 nm, 1684-1685 nm, 1693.5-1695.5 nm, 1700-1705 nm, 1708.5-1710 nm	1692-1706 nm, 1816-1844 nm, 1948-2018 nm	1104 nm, 1108-1116 nm, 1122 nm, 1448 nm, 1458-1460 nm, 1484-1486 nm, 1492-1494 nm, 1498 nm, 1502-1504 nm, 1510-1512 nm, 1520 nm, 1524-1530 nm, 1538 nm, 1578-1580 nm, 1586 nm, 1594-1596 nm
Fructose	2244-2244.5 nm, 2246.5 nm, 2269.5-2271 nm, 2275.5-2279.5 nm, 2285.5-2289 nm, 2304.5-2305.5 nm, 2318-2321 nm	1794-1846 nm, 1880-1930 nm, 1942-1990 nm, 2004-2044 nm, 2234-2298 nm, 2348-2384 nm, 2432-2460 nm	1522-1534 nm
Reducing sugars	1934-1937.5 nm, 2045-2053.5 nm, 2236.5-2237 nm, 2243.5-2246 nm, 2264-2269 nm, 2310.5-2322 nm, 2334.5-2347 nm	1948 nm, 1958-1964 nm, 1976-1990 nm, 2028-2030 nm, 2068-2074 nm, 2126-2128 nm, 2154-2164 nm, 2276-2284 nm	1464-1600 nm
pH	1030-1032 nm, 1049.5-1050 nm, 1058-1061 nm, 1228-1230 nm, 1234.5-1237.5 nm, 1272-1273 nm, 1279-1280 nm, 1282.5-1283 nm, 1698.5-1700.5 nm, 1707-1707.5 nm	2376-2456 nm	1108 nm, 1112-1114 nm, 1122 nm, 1168-1178 nm, 1182 nm, 1186-1196 nm, 1202 nm, 1216 nm, 1570-1574 nm, 1582-1592 nm, 1596-1598 nm

Botanical Origin	1761.5-1771 nm, 1809.5-1816.5 nm	2444-2470 nm	1576-1600 nm
